# Geriatric assessment–integrated prediction model for postoperative deep vein thrombosis in older adults undergoing transurethral resection of the prostate: retrospective cohort study

**DOI:** 10.3389/fmed.2026.1899303

**Published:** 2026-08-06

**Authors:** Jiaran Zhang, Yuru Yan, Wei Zhou, Hanyu Xin

**Affiliations:** 1 Department of Nursing, Renji Hospital, School of Medicine, Shanghai Jiao Tong University, Shanghai, China; 2 Department of Urology, Renji Hospital, School of Medicine, Shanghai Jiao Tong University, Shanghai, China; 3 Department of Neurology, Punan Branch of Renji Hospital, Shanghai Jiaotong University School of Medicine (Punan Hospital in Pudong New District, Shanghai), Shanghai, China

**Keywords:** clinical decision support systems, deep vein thrombosis, geriatric assessment, prostatic hyperplasia, risk assessment, transurethral resection of prostate

## Abstract

**Background:**

Postoperative deep vein thrombosis (DVT) is an important complication in older patients undergoing transurethral resection of the prostate (TURP). Although associations between geriatric vulnerability and thrombosis are established, these domains are not routinely integrated into TURP-specific risk assessment. We therefore evaluated whether preoperative geriatric assessment (GA) domains add predictive information to age-adjusted D-dimer in this setting.

**Methods:**

In this retrospective single-center cohort (*n* = 420; 294 development and 126 internal validation), an exploratory two-stage procedure comprising univariable screening followed by LASSO regression identified four predictors: preoperative age-adjusted D-dimer positivity, serum albumin, Activities of Daily Living (ADL) score, and Mini-Mental State Examination (MMSE) score. An unweighted logistic regression model was used as the primary integrated model. Discrimination, calibration, precision-recall performance, and decision-curve analysis were assessed. Logistic regression (LR), random forest (RF), and support vector machine (SVM) were additionally compared using the same development-set cross-validated sigmoid calibration framework.

**Results:**

The perioperative DVT incidence was 11.19% (47/420). In the internal validation cohort, the primary integrated LR model achieved an AUC of 0.750 (95% CI: 0.557–0.903), a precision-recall AUC of 0.457, and a Brier score of 0.081. Compared with the D-dimer-only clinical model, the integrated model showed improvements in NRI (0.893, 95% CI: 0.360–1.345; *P* < 0.001) and IDI (0.078, 95% CI: 0.020–0.145; *P* = 0.015). At a prespecified probability threshold of 0.20, sensitivity was 0.571 and specificity was 0.911. In the uniformly calibrated algorithm comparison, SVM had the highest AUC point estimate (0.796), but its difference from LR (0.751) was not statistically conclusive (bootstrap difference 0.045, 95% CI: −0.004 to 0.102; *P* = 0.071).

**Conclusions:**

A TURP-specific model combining preoperative age-adjusted D-dimer with geriatric assessment domains showed preliminary value for postoperative DVT risk stratification. Because the study was retrospective, single-center, and based on only 47 events, the findings should be considered exploratory and require prospective external validation before clinical implementation.

## Introduction

Transurethral resection of the prostate (TURP) remains the standard surgical intervention for benign prostatic hyperplasia (BPH). The growing burden of BPH in aging populations has established older men as a predominant population undergoing this procedure (1). Despite surgical advances, venous thromboembolism, including DVT, remains a clinically important postoperative complication after TURP (2). Given the advanced age and pronounced physiological heterogeneity of elderly TURP patients, postoperative thromboprophylaxis frequently encounters a clinical dilemma: the delicate balance between thrombosis due to under-anticoagulation and hemorrhagic complications from over-anticoagulation. Consequently, precise DVT risk stratification is paramount for optimizing individualized prophylactic anticoagulant regimens and minimizing bleeding complications.

Traditional DVT risk assessment models rely primarily on routine clinical parameters, with factors such as prior VTE and postoperative bladder hematoma reported as risk factors after TURP (2). Although conventional biochemical markers like D-dimer are central to thrombosis evaluation, their prognostic value is significantly compromised in the elderly due to age-related physiological elevations. Age-adjusted thresholds can improve diagnostic specificity in older adults, although their perioperative use requires separate validation (3, 4). Even so, existing models fail to address the specific characteristics of the elderly TURP population by systematically integrating aging-related functional and physiological decline indicators. Relying solely on the aforementioned clinical and biochemical parameters is insufficient to capture the comprehensive thrombosis risk profile of this vulnerable cohort.

Existing evidence links functional dependence, cognitive impairment, hypoalbuminemia, and medication burden with thrombotic risk in older or surgical populations (5–8). The novelty of the present study is therefore not the identification of these individual associations, but their combined evaluation with age-adjusted D-dimer in a model tailored to older patients undergoing TURP. Whether this integration improves TURP-specific risk stratification remains uncertain.

Accordingly, the primary objective of this study was to develop and internally evaluate an exploratory TURP-specific prediction model that integrates preoperative GA domains with age-adjusted D-dimer. Secondary objectives were to quantify the incremental value of the integrated model over a D-dimer-only model, compare representative algorithms under a common calibration framework, and present a research-use web calculator prototype for transparent probability estimation.

## Methods

### Study design

In this retrospective cohort study, a total of 846 patients who underwent elective transurethral resection of the prostate (TURP) for benign prostatic hyperplasia (BPH) at Renji Hospital, Shanghai Jiao Tong University School of Medicine, between February 2023 and March 2026, were consecutively screened. Following a rigorous screening process based on predefined inclusion and exclusion criteria, 420 patients were ultimately enrolled in the final analysis.

### Ethical considerations

This study was approved by the Institutional Review Board (IRB) of Renji Hospital, Shanghai Jiao Tong University School of Medicine (Approval No: LY2026-108-B) and adhered to the Declaration of Helsinki. The requirement for informed consent was waived due to the retrospective nature of the research. To protect participant privacy, all data were deidentified and stored securely. No participant compensation was provided.

### Selection criteria

To ensure the homogeneity of the cohort and minimize confounding effects on coagulation and geriatric parameters, strict selection criteria were applied.

**Inclusion criteria:** (1) Confirmed diagnosis of BPH with surgical indications for elective TURP; (2) Age ≥ 65 years, validating the necessity of geriatric assessments; (3) Availability of complete preoperative Geriatric Assessment (GA) data, comprising four key domains: cognitive function (Mini-Mental State Examination, MMSE), functional status (Activities of Daily Living, ADL), nutritional status (serum albumin levels), and polypharmacy status (defined as the concurrent use of ≥ 5 chronic medications); (4) Adherence to a standardized preoperative screening protocol: all patients with a preoperative D-dimer level > 0.5 mg/L must have undergone lower extremity compression ultrasonography (CUS) prior to surgery, with no pre-existing DVT detected; (5) Complete documentation of postoperative CUS results for the diagnosis of incident DVT.

**Exclusion criteria**: (1) A history of venous thromboembolism (VTE) or preoperative CUS indicating pre-existing DVT; (2) Diagnosis of prostate cancer or other active systemic malignancies; (3) Known primary coagulopathy or severe hepatic/renal insufficiency; (4) Regular administration of therapeutic anticoagulants or antiplatelet agents within 2 weeks prior to surgery; (5) Recent severe acute physiological stress within the past 3 months (e.g., major trauma, sepsis); (6) Severe pre-existing psychiatric disorders precluding accurate clinical evaluation and GA scoring.

### Data collection and variable definition

#### Clinical characteristics and laboratory measurements

Baseline clinical and perioperative data were extracted from the electronic medical record. The primary prediction model used information available preoperatively: age, preoperative D-dimer, serum albumin, ADL score, and MMSE score. For patients older than 50 years, the age-adjusted D-dimer cutoff was age × 0.01 mg/L (3, 4); values above the individual cutoff were classified as positive. Postoperative bladder hematoma and postoperative D-dimer were recorded for descriptive or separate diagnostic-triage analyses and were not used as predictors in the preoperative model.

#### Preoperative geriatric assessment

To evaluate physiological and functional reserve, a comprehensive GA was completed within 48 h before surgery. Functional status was assessed using the Activities of Daily Living (ADL) scale (Barthel Index), reflecting the degree of physical independence. Cognitive function was measured using the Mini-Mental State Examination (MMSE; range 0–30), with lower scores indicating poorer cognition. Nutritional status was represented by the preoperative serum albumin level, as detailed in the laboratory measurements. Medication burden was assessed as polypharmacy, defined as the regular use of five or more chronic medications at admission. Polypharmacy was treated as an indicator of medication burden and clinical complexity rather than as a direct measurement of comorbidity (5).

#### Assessment of postoperative DVT

The primary endpoint was the occurrence of incident postoperative DVT. Following the clinical protocol, all participants underwent lower extremity compression ultrasonography (CUS) between postoperative days 2 and 4, or earlier if symptoms such as unilateral limb swelling or pain occurred. DVT was diagnosed based on the presence of a non-compressible venous segment or intraluminal thrombus on B-mode imaging. Examinations were performed by two senior vascular sonographers blinded to the patients' preoperative data.

### Postoperative thromboprophylaxis

All patients received standardized postoperative thromboprophylaxis consisting of early mobilization and intermittent pneumatic compression. Routine pharmacological prophylaxis was not administered because of the procedure-related bleeding risk; anticoagulation was considered individually when clinically indicated.

### Variable selection and model development

Candidate reduction used a two-stage procedure in the development cohort. Univariable logistic regression was first applied using *P* < 0.10 as a screening criterion. Variables retained after univariable screening were assessed for multicollinearity using variance inflation factors (VIFs), and variables without marked multicollinearity were then entered into LASSO regression with 10-fold cross-validation.

Two logistic regression models were developed: a clinical model containing preoperative age-adjusted D-dimer positivity alone and an integrated model containing age-adjusted D-dimer positivity, serum albumin, ADL score, and MMSE score. The final integrated LR model used native probabilities for the main performance, reclassification, nomogram, and risk-stratification analyses. In a separate algorithm comparison, LR, RF, and SVM were selected to represent an interpretable linear model, a tree-based ensemble, and a kernel-based nonlinear method, respectively. All three algorithms were evaluated using the same five-fold development-set cross-validated sigmoid calibration procedure; balanced class weights were applied only to RF and SVM. The internal validation cohort was not used for model fitting, hyperparameter selection, calibration fitting, or threshold selection.

### Model interpretation and web application

Because the selected primary model was logistic regression, interpretation was based on regression coefficients, odds ratios, confidence intervals, and the nomogram.

A research-use web calculator prototype was developed from the final logistic regression equation. The interface accepts preoperative age-adjusted D-dimer status, serum albumin, ADL score, and MMSE score and returns an estimated probability. The calculator is intended for transparent illustration and requires external validation before clinical use.

### Statistical analysis and evaluation

All computational analyses and model development were primarily implemented using Python (version 3.9; Python Software Foundation, Wilmington, DE, USA), utilizing libraries such as scikit-learn, pandas, and statsmodels, supplemented by IBM SPSS Statistics (version 26.0; IBM Corp., Armonk, NY, USA) for basic descriptive statistics. Continuous variables were assessed for normality using the Shapiro-Wilk test and presented as mean ± standard deviation (SD) or medians with interquartile ranges (IQR). Comparisons were conducted using the independent samples *t*-test or Mann-Whitney *U* test. Categorical variables were expressed as frequencies (%) and analyzed using the Chi-square test or Fisher's exact test.

Model performance in the internal validation cohort was evaluated using ROC-AUC with 95% confidence intervals, precision-recall AUC, Brier score, calibration plots, calibration intercept and slope, and decision-curve analysis. Sensitivity, specificity, positive predictive value, negative predictive value, F1 score, and balanced accuracy were reported at the conventional 0.50 threshold and at a screening-oriented 0.20 threshold. Exploratory low-, intermediate-, and high-risk strata were defined using probability boundaries of < 0.25, 0.25–0.60, and ≥0.60, respectively. These strata were used for descriptive risk separation and were not intended as treatment thresholds. NRI and IDI were retained as secondary exploratory measures of incremental value.

Given the imbalanced outcome distribution, precision-recall AUC and threshold-dependent measures were emphasized alongside ROC-AUC. For the three-algorithm comparison, paired bootstrap resampling was used to quantify the AUC difference between SVM and LR. Decision-curve net benefit was reported at clinically interpretable thresholds rather than relying only on a summary area measure.

The final model contained four predictor parameters and the development cohort included 33 DVT events, corresponding to 8.25 events per predictor; the full cohort contained 47 events (11.75 events per predictor). A scenario-based minimum sample-size assessment was conducted using the Riley framework for binary-outcome prediction models (9), assuming four predictor parameters, an outcome proportion of 0.112, and a target global shrinkage factor of 0.90. With an anticipated Cox–Snell *R*^2^ of 0.109, the estimated minimum sample size was approximately 310 participants; under a more conservative *R*^2^ of 0.08, it was approximately 430. The actual development cohort (*n* = 294) was slightly below the primary estimate and clearly below the conservative estimate; therefore, the available sample size was considered limited and the model results were interpreted as exploratory.

### Missing data handling

**Figure 1:**
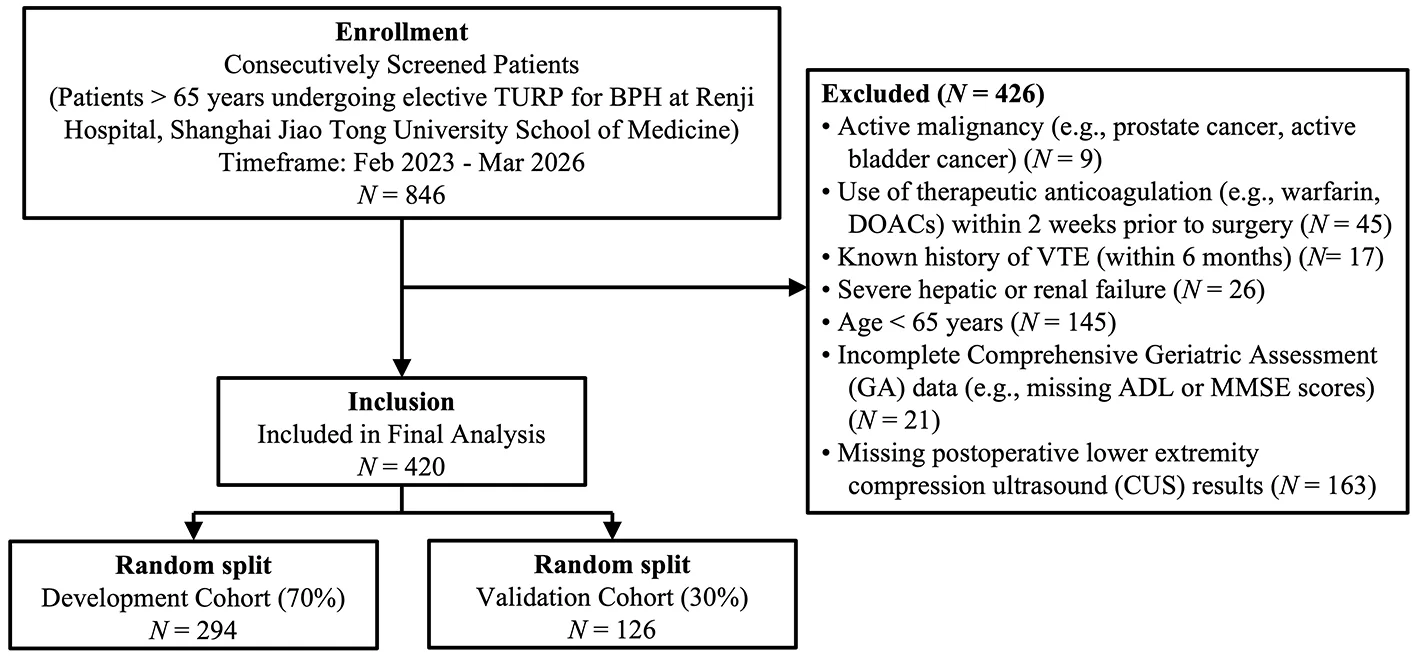
Flowchart of participant screening and enrollment. Of 846 consecutive patients screened between February 2023 and March 2026, 426 were excluded based on the following criteria: age < 65 years (*n* = 145), active malignancy (*n* = 9), preoperative therapeutic anticoagulation (*n* = 45), prior history of VTE (*n* = 17), severe organ failure (*n* = 26), incomplete GA data (*n* = 21), and missing postoperative ultrasound (*n* = 163). The remaining 420 patients were randomly split into the development cohort (*n* = 294, 70%) and internal validation cohort (*n* = 126, 30%). BPH, benign prostatic hyperplasia; TURP, transurethral resection of the prostate; VTE, venous thromboembolism; GA, comprehensive geriatric assessment; CUS, compression ultrasound.

The screening flow and reasons for exclusion are shown in Figure 1. A complete-case analytic cohort was constructed from patients with complete preoperative GA data and an evaluable postoperative ultrasound outcome. No predictor or outcome values were missing in the final analytic dataset (Supplementary Table 5).

### Transparent reporting

This study was conducted and reported in accordance with the updated TRIPOD+AI statement for prediction models developed using regression or machine-learning methods (10).

## Results

### Patient characteristics

A total of 420 older patients who underwent TURP were included in the final analytic cohort. No values were missing for the variables used in the final modeling dataset; baseline clinical variables, GA indicators, laboratory biomarkers, and postoperative ultrasound outcomes were documented for all included patients. The overall perioperative incidence of DVT was 11.19% (47/420). Using a random 70:30 split stratified by DVT status, the cohort was partitioned into a development set for model construction (*n* = 294; 33 events) and an internal validation set for performance evaluation (*n* = 126; 14 events) (Figure 1).

Baseline comparisons between the development and internal validation sets showed broadly similar demographic characteristics, perioperative clinical indicators, laboratory biomarkers, and GA domains, including ADL and MMSE scores (all *P* > 0.05; Table 1). These findings support approximate balance between the two internal cohorts, but the absence of statistically significant differences should not be interpreted as proof of equivalence, and the validation set remained an internal holdout sample from the same center and period.

**Table 1:** Baseline characteristics and comparison of the development and internal validation cohorts.

Variables	Total (*N* = 420)	Development cohort (*n* = 294)	Internal validation cohort (*n* = 126)	*P*
Age (years)	72.0 (69.0–76.0)	72.0 (69.0–76.0)	72.0 (69.0–75.0)	0.762
ADL score	88.0 (79.2–94.0)	88.0 (79.0–94.0)	89.8 (79.3–93.9)	0.540
MMSE score	26.0 (24.0–27.0)	26.0 (24.0–27.0)	25.0 (24.0–27.0)	0.423
Serum albumin (g/L)	38.41 ± 3.09	38.45 ± 3.16	38.31 ± 2.94	0.666
Cognitive impairment, *n* (%)	145 (34.5%)	96 (32.7%)	49 (38.9%)	0.263
Polypharmacy (≥5 drugs), *n* (%)	198 (47.1%)	139 (47.3%)	59 (46.8%)	> 0.99
Postoperative hematoma, *n* (%)	48 (11.4%)	35 (11.9%)	13 (10.3%)	0.763
Preoperative d-dimer (mg/L)	0.52 (0.33–0.77)	0.53 (0.33–0.73)	0.51 (0.34–0.85)	0.671
Age-adjusted cut-off (mg/L)	0.72 (0.69–0.76)	0.72 (0.69–0.76)	0.72 (0.69–0.75)	0.762
Age-adjusted d-dimer Positive^a^, *n* (%)	108 (25.7%)	73 (24.8%)	35 (27.8%)	0.609
Incidence of DVT, *n* (%)	47 (11.2%)	33 (11.2%)	14 (11.1%)	> 0.99

Continuous variables are presented as mean ± SD for normally distributed data and median (IQR) for non-normally distributed data. Categorical variables are presented as frequencies and percentages [*n* (%)]. *P* < 0.05 indicates statistical significance. ADL, activities of daily living; MMSE, mini-mental state examination; VTE, venous thromboembolism; DVT, deep vein thrombosis; IQR, interquartile range; SD, standard deviation. ^a^The age-adjusted cut-off was calculated as age × 0.01 mg/L for patients older than 50 years, based on the ADJUST-DVT study (3). A test result higher than this individual cut-off was defined as ‘positive'.

### Feature selection and multivariable analysis

Univariable logistic regression was first performed in the development cohort using *P* < 0.10 as the screening threshold. Five variables met this criterion: age-adjusted D-dimer positivity, serum albumin, ADL score, MMSE score, and cognitive impairment.

**Figure 2:**
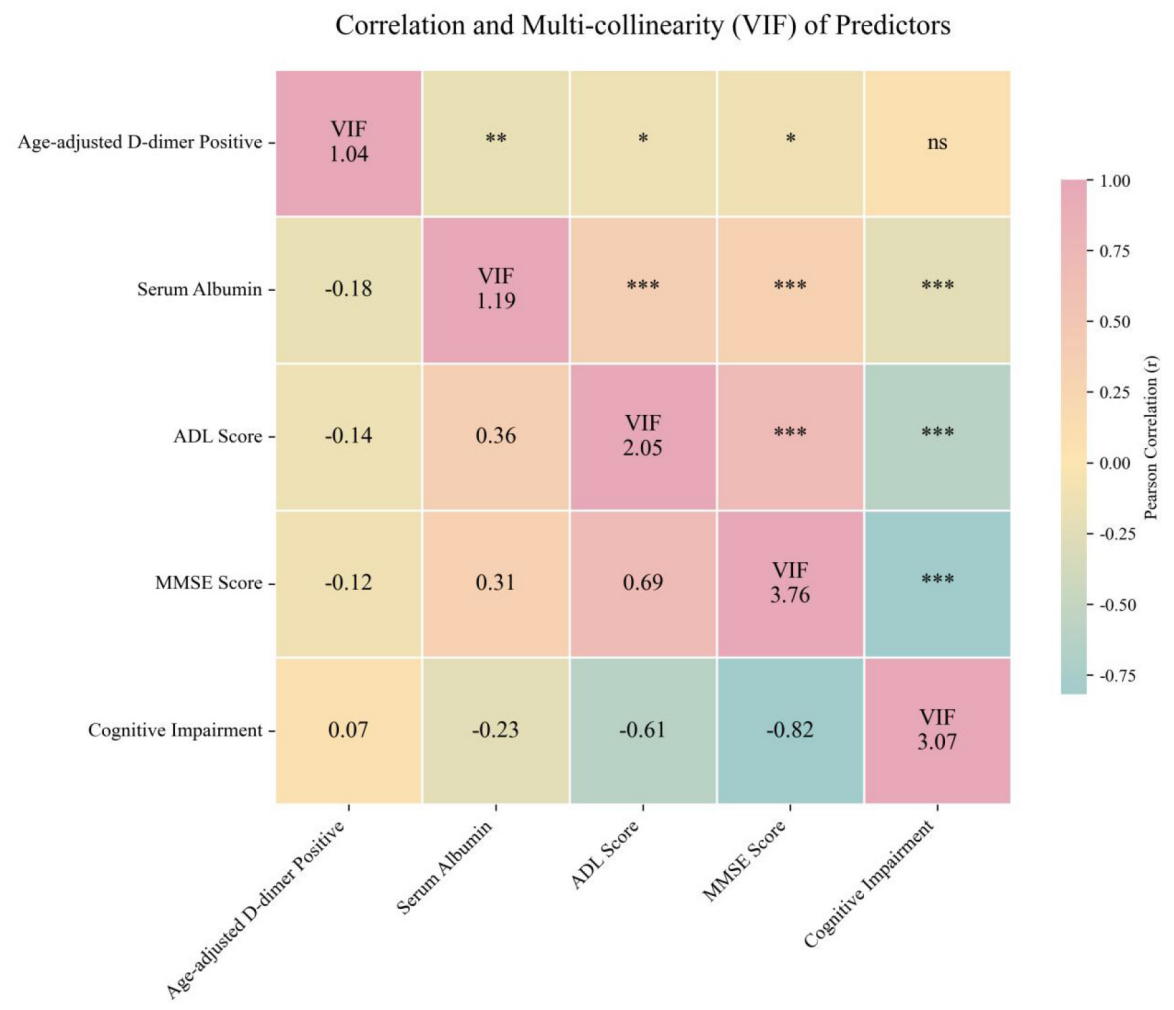
Integrated heatmap of correlation and multi-collinearity analysis. The square matrix presents a multidimensional evaluation of the candidate predictors. The diagonal elements display the Variance Inflation Factor (VIF) for each variable to assess multi-collinearity, where values of inf denote perfect collinearity. The lower triangular matrix shows the Pearson correlation coefficients (*r*) combined with significance levels. The upper triangular matrix exclusively presents the significance levels indicated by asterisks (*P < 0.05, **P < 0.01, ***P < 0.001, ns, not significant). This hybrid visualization demonstrates the intrinsic functional interconnectedness within Geriatric Assessment (GA) domains while confirming the absence of severe multi-collinearity among the candidate predictors screened via univariate analysis (all VIFs < 5).

**Figure 3:**
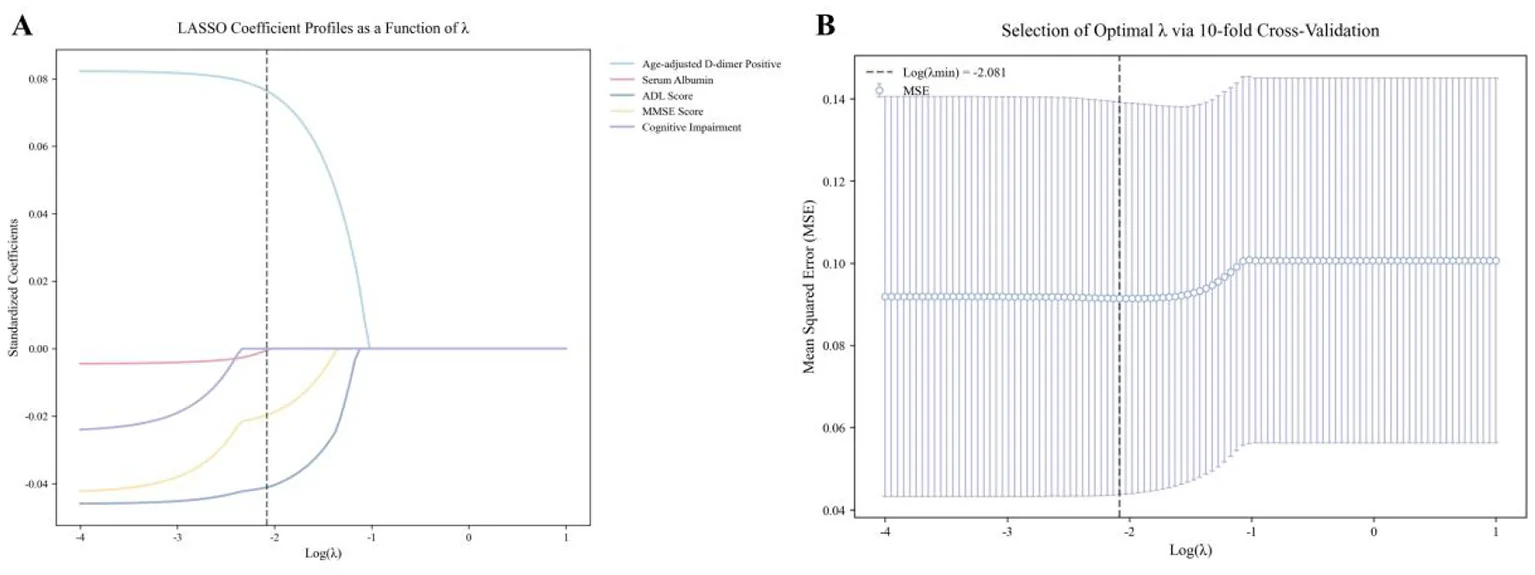
Predictor selection process using the LASSO regression model. **(A)** LASSO coefficient profiles of the candidate predictors as a function of log10(λ). Each colored line represents the coefficient path of an individual variable; as the penalty intensity increases, less informative variables are compressed toward zero. **(B)** Selection of the optimal tuning parameter (λ) via 10-fold cross-validation. The vertical dashed line indicates the optimal value (λ min = 0.0083, log10(λ) = −2.081), where the mean squared error is minimized. At the optimal log10(λ), four key variables with non-zero coefficients were retained: age-adjusted D-dimer positivity, serum albumin, ADL score, and MMSE score.

Before LASSO regression, multicollinearity among these five candidates was assessed using VIFs. VIF values ranged from 1.043 to 3.763, indicating no marked multicollinearity (Figure 2). Using 10-fold cross-validation (λ = 0.0083), LASSO retained four preoperative predictors: age-adjusted D-dimer positivity, serum albumin, ADL score, and MMSE score (Figure 3). These predictors were carried forward to the multivariable logistic regression model.

In the multivariable model, age-adjusted D-dimer positivity was associated with higher odds of postoperative DVT (OR 5.160, 95% CI: 2.327–11.442; *P* < 0.001). Serum albumin, ADL score, and MMSE score were retained as model predictors but were not individually statistically significant (Table 2). Their coefficients were −0.013, −0.045, and −0.106, respectively.

**Table 2:** Multivariable logistic regression predictor estimates for postoperative DVT.

Variables	β (Coef)	OR (95% CI)	*P*
Constant (intercept)	4.227	-	0.128
Clinical indicators			
Age-adjusted d-dimer positive	1.641	5.160 (2.327–11.442)	< 0.001
Geriatric assessment (GA)			
ADL score	−0.045	0.956 (0.901–1.014)	0.132
MMSE score	−0.106	0.900 (0.714–1.133)	0.369
Serum albumin	−0.013	0.987 (0.862–1.131)	0.852

The multivariable model was constructed using variables selected via LASSO regression. All categorical variables were entered as binary factors. *P* < 0.05 indicates statistical significance. The regression coefficients derived here served as the foundational parameters for the integrated model. The primary integrated LR analyses used native probabilities; the separate uniformly calibrated algorithm comparison is described in Methods and Supplementary Table 2. OR, odds ratio; CI, confidence interval; ADL, activities of daily living; VTE, venous thromboembolism.

### Construction and performance evaluation of the clinical baseline predictive model

To evaluate the baseline efficacy of a single conventional clinical indicator in predicting DVT risk, we first constructed a clinical logistic regression model incorporating only the Age-adjusted D-dimer. This model was developed without any class-weight intervention to authentically reflect the discriminative capacity of a commonly used clinical screening marker.

**Figure 4:**
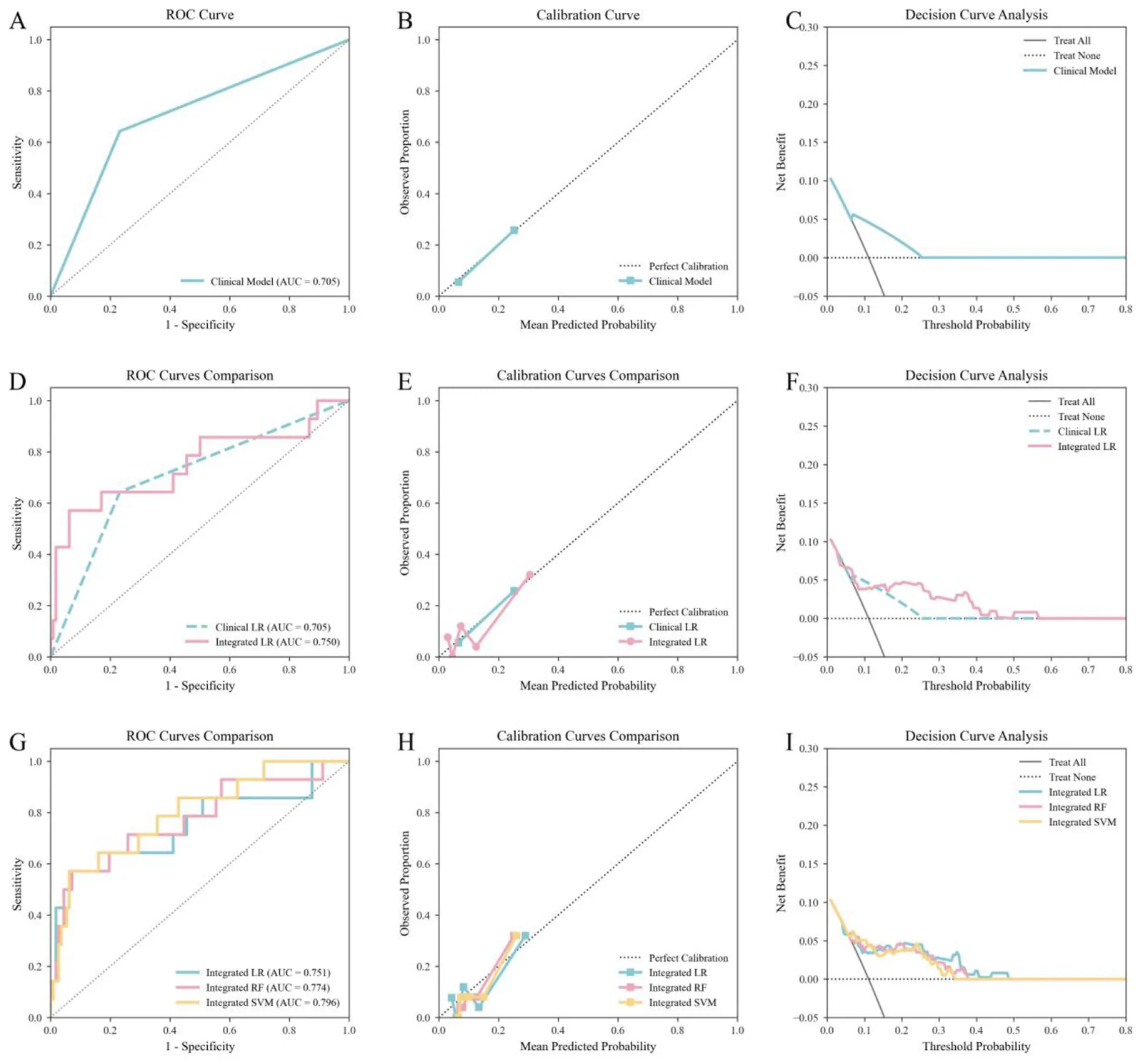
Evaluation and comparison of DVT prediction models. **(A-F)** Show the primary native-probability clinical and integrated LR models; **(G-I)** Show the separate uniformly calibrated three-algorithm comparison. (1) Evaluation of the clinical model for DVT prediction. **(A)** ROC curve: the stepwise trajectory reflects the limited probability space provided by a single dichotomous variable (Age-adjusted D-dimer). **(B)** Calibration curve: The calibration curve is shown for descriptive assessment of agreement between predicted and observed probabilities; calibration uncertainty is substantial given the limited number of validation events. **(C)** DCA: the net benefit curve shows a narrow clinical utility window, dropping sharply above a 0.15 threshold, highlighting the limitations of relying solely on traditional markers for individualized assessment. (2) Evaluation of the integrated model incorporating Geriatric Assessment (GA). **(D)** ROC curves: compared to the clinical model, the integrated model exhibits a smoother, more expansive trajectory, with the AUC increasing to 0.750 (95% CI: 0.557–0.903). **(E)** Calibration curves: the Brier Score for the integrated model was 0.081; calibration and clinical utility should be interpreted cautiously given the limited validation sample. **(F)** DCA: the integrated model showed threshold-dependent net benefit relative to the clinical model. (3) Uniformly calibrated algorithm comparison. **(G)** ROC curves: validation AUC was 0.751 for LR, 0.774 for RF, and 0.796 for SVM. **(H)** Calibration curves show the observed-versus-predicted relationship for each algorithm after five-fold development-set cross-validated sigmoid calibration. **(I)** Decision curves show threshold-dependent net benefit; no algorithm was uniformly superior. AUC, area under the ROC curve; LR, logistic regression; RF, random forest; SVM, support vector machine; DCA, decision curve analysis. **(A-F)** Use the native probabilities from the primary unweighted LR analyses. For **(G-I)**, all three algorithms underwent the same five-fold development-set cross-validated sigmoid calibration; balanced class weights were used only for RF and SVM. The internal validation cohort was used only for final evaluation.

The evaluation results (Supplementary Table 1) showed that the clinical baseline model achieved an AUC of 0.701 (95% CI: 0.610–0.789) in the development set and 0.705 (95% CI: 0.561–0.835) in the validation set. Although the model exhibited some discriminative potential, its sensitivity was 0.000 in both datasets, with a specificity of 1.000, indicating that driven by a single indicator, the model tended to classify all subjects as non-DVT, leading to a significant risk of underdiagnosis. The Brier score (0.091) further confirmed the limitations of this model in terms of probability prediction accuracy (Figures 4A, B).

Quantitative Decision Curve Analysis (DCA) demonstrated limited clinical utility for the clinical baseline model (Figure 4C), with an Area Under the Decision Curve (AUDC) of only 0.0101 in the validation set. At a common clinical risk threshold of 20%, the Net Benefit (NB) was only 0.0205. This marginal clinical utility highlights the insufficiency of relying solely on D-dimer for DVT risk early warning in elderly perioperative patients, thereby justifying the necessity of incorporating multi-dimensional GA features to construct an integrated predictive model.

### Development and systematic optimization of the integrated predictive model

#### Comparison between integrated and clinical models and assessment of incremental value

To evaluate the incremental value of geriatric assessment (GA) domains, we compared the D-dimer-only clinical model with the integrated logistic regression (LR) model. In the internal validation cohort, AUC increased from 0.705 (95% CI: 0.561–0.835) for the clinical model to 0.750 (95% CI: 0.557–0.903) for the integrated model. The lower Brier score for the integrated model (0.081 vs. 0.091) indicated lower overall probabilistic prediction error; calibration should be interpreted cautiously in view of the limited internal validation sample (Figures 4D–F).

Continuous reclassification indices were higher after addition of the GA domains: NRI was 0.893 (95% CI: 0.360–1.345; *P* < 0.001) and IDI was 0.078 (95% CI: 0.020–0.145; *P* = 0.015). These exploratory measures should be interpreted cautiously given the limited number of validation events. At the conventional 0.50 threshold, the integrated model had sensitivity of 0.071 and specificity of 0.991 (Table 3).

**Table 3:** Performance metrics and incremental value indices of the integrated LR vs. clinical baseline model.

Metrics	Clinical model (d-dimer)	Integrated model (d-dimer + GA)	*P*-value
AUC [95% CI]	0.705 [0.561–0.835]	0.750 [0.557–0.903]	-
Brier score	0.091	0.081	-
Sensitivity	0.000	0.071	-
Specificity	1.000	0.991	-
NRI [95% CI]	Reference	0.893 [0.360–1.345]	< 0.001
IDI [95% CI]	Reference	0.078 [0.020–0.145]	0.015

The Clinical Model was constructed using only the age-adjusted D-dimer positive status as a single predictor. The Integrated Model incorporated four variables: age-adjusted D-dimer, serum albumin, ADL score, and MMSE score. The 95% CIs for AUC and reclassification indices were estimated using the bootstrapping method with 1,000 resamples. NRI (continuous) and IDI were calculated to quantify the incremental value of adding GA features to the clinical baseline. A positive NRI/IDI with *P* < 0.05 indicates a significant improvement in the model's ability to correctly reclassify or discriminate patients' risk levels. Both models in this comparison were developed using standard LR without class-weight adjustment to emphasize the raw discriminative contribution of the added variables. AUC, area under the receiver operating characteristic curve; CI, confidence interval; LR, logistic regression; GA, geriatric assessment; NRI, net reclassification improvement; IDI, integrated discrimination improvement.

#### Identification of the optimal algorithmic model for the integrated predictive model

The separate uniformly calibrated algorithm comparison is summarized in Supplementary Table 2 and Figures 4G–I.

In the uniformly calibrated comparison, validation ROC-AUC values were 0.751 for LR, 0.774 for RF, and 0.796 for SVM, while precision-recall AUC values were 0.467, 0.472, and 0.437, respectively (Supplementary Table 2; Figures 4G, H; Supplementary Figure 4). The paired bootstrap AUC difference between SVM and LR was 0.045 (95% CI: −0.004 to 0.102; *P* = 0.071), providing insufficient evidence that SVM discriminated better than LR.

Decision-curve analysis showed small and threshold-dependent differences among algorithms. At a 10% threshold, net benefit was 0.035 for LR, 0.037 for RF, and 0.050 for SVM; at a 20% threshold, corresponding values were 0.046, 0.046, and 0.038. No algorithm was uniformly superior across the evaluated thresholds.

LR was retained as the primary model because its discrimination was not conclusively inferior to SVM, its precision-recall performance was comparable, and its coefficients and nomogram permit direct clinical interpretation. The algorithm comparison should be viewed as exploratory rather than as evidence of superiority.

For the final integrated LR model used in the main analyses (native probabilities, before the separate algorithm-comparison calibration), validation precision-recall AUC was 0.457 and the Brier score was 0.081. At the conventional 0.50 threshold, sensitivity was only 0.071 despite high specificity (0.991), indicating poor screening performance. At the screening-oriented 0.20 threshold, sensitivity increased to 0.571 while specificity remained 0.911; positive predictive value was 0.444, F1 score was 0.500, and balanced accuracy was 0.741 (Supplementary Table 4). These findings support using lower probability thresholds for surveillance-oriented risk enrichment rather than relying on the conventional 0.50 threshold.

#### Construction of the integrated nomogram

An individualized nomogram was constructed from the final integrated logistic regression equation (Supplementary Figure 1). Point allocation reflects the fitted coefficients and predictor ranges. Given the limited number of events and lack of external validation, the nomogram should be considered a research tool rather than a validated clinical score.

### Clinical risk stratification

Exploratory risk strata were evaluated in the internal validation cohort using the probability boundaries defined in the Methods. Observed DVT incidence was 5.13%, 6.35%, and 33.33% in the low-, intermediate-, and high-risk groups, respectively (Table 4). These categories showed descriptive risk separation within this dataset but should not be interpreted as validated treatment thresholds.

**Table 4:** Predictive performance of the model across risk strata (internal validation cohort).

Risk level	Probability threshold	Total patients (*N*)	DVT cases (*n*)	Observed incidence (%)	Relative risk (95% CI)
Low risk	< 0.25	39	2	5.13	1.00 (Ref.)
Moderate risk	0.25–0.60	63	4	6.35	1.24 [0.24–6.44]
High risk	≥0.60	24	8	33.33	6.50 [1.53–27.56]

Relative Risk (RR) was calculated using the low-risk category as the reference group. DVT, deep vein thrombosis; CI, confidence interval.

### Diag**nostic triaging and resource optimization**

Postoperative D-dimer levels are universally elevated due to surgical trauma and tissue repair, significantly compromising the specificity of the traditional fixed threshold (>0.5 mg/L) for diagnosing Deep Vein Thrombosis (DVT). To optimize the allocation of postoperative ultrasound (US) resources, we compared the diagnostic efficacy of the Age-adjusted D-dimer (postoperative) strategy against the traditional fixed threshold strategy within the validation cohort (*N* = 126).

Results demonstrated that while the traditional strategy maintained a sensitivity of 1.00, its specificity was limited to 0.73 with a low positive predictive value (PPV) of 0.32, suggesting a high rate of false positives and unnecessary US examinations in clinical practice. In contrast, the age-adjusted strategy significantly improved specificity to 0.97 (*P* < 0.001) and optimized PPV to 0.81. Notably, there was no statistically significant decrease in sensitivity (0.93 vs. 1.00, *P* > 0.99), while maintaining a high negative predictive value (NPV) of 0.99 (Supplementary Table 3, Supplementary Figure 3).

Resource optimization assessment revealed that the traditional strategy would require US examinations for 44 patients, whereas the age-adjusted strategy reduced this requirement to only 16 patients. This indicates that applying the age-adjusted criteria in postoperative monitoring could circumvent 63.64% of unnecessary US screenings. This analysis confirms that the Age-adjusted D-dimer serves as a highly efficient and precise postoperative triaging marker, significantly alleviating the burden on limited healthcare resources. This optimization is particularly crucial in high-volume surgical centers where ultrasound resources are often constrained.

## Discussion

This study evaluated an exploratory TURP-specific approach that combines preoperative age-adjusted D-dimer with functional, cognitive, and nutritional measures. The main contribution is the integration of established geriatric risk domains within a TURP cohort, rather than the discovery of previously unknown associations. The integrated model showed moderate discrimination and improved exploratory reclassification indices relative to D-dimer alone, but its limited sensitivity at conventional thresholds and the small number of events constrain immediate clinical use.

Existing urologic VTE assessment approaches include the general Caprini score, Caprini-derived machine-learning work in urologic inpatients, and recent nomograms for mixed urologic procedures (11–14). In addition, the European Association of Urology (EAU) and American Urological Association (AUA) VTE risk-assessment approaches are commonly used in urologic practice, providing closely aligned recommendations for thromboprophylaxis and practical clinical application (15). However, these urology-specific risk-assessment approaches were not developed specifically for TURP and do not directly incorporate the geriatric functional, cognitive, nutritional, and age-adjusted D-dimer features evaluated here. Although they provide valuable perioperative risk context, they may rely on broad risk categories or postoperative variables and do not integrate multidimensional geriatric assessment with preoperative age-adjusted D-dimer. In contrast, the present model was developed for a narrowly defined TURP cohort, used protocolized ultrasound outcome assessment, relied on four variables available before surgery, and produced an individualized probability through a transparent LR equation. It should therefore be viewed as a complementary TURP-specific research tool rather than a replacement for established VTE risk-assessment models.

Postoperative D-dimer is frequently elevated after surgery, reducing the specificity of a conventional fixed cutoff. In the present internal validation cohort, the age-adjusted strategy reduced the number of ultrasound referrals from 44 to 16 and increased specificity from 0.732 to 0.973, while sensitivity decreased from 1.000 to 0.929. This separate diagnostic-triage analysis suggests a potential reduction in unnecessary imaging, but the single false-negative result and small number of DVT events require prospective safety evaluation before implementation.

The selected geriatric domains have plausible clinical relevance: impaired mobility may reduce calf-muscle pump function, cognitive impairment may limit mobilization and symptom reporting, and lower albumin may reflect frailty, inflammation, or poor nutritional reserve (16–20). These mechanisms provide biological rationale for evaluating the variables together, but the present observational analysis did not test interactions or establish causal synergy.

A potential future pathway is to use a validated preoperative model to identify patients who may benefit from closer postoperative surveillance while separately applying postoperative D-dimer as a diagnostic triage tool. The current results do not establish thresholds for pharmacological prophylaxis, particularly because TURP requires balancing thrombotic risk against procedure-related bleeding risk (17).

Several limitations warrant emphasis. First, the retrospective single-center design, complete-case inclusion, and only 47 DVT events limit precision and generalizability. Second, the two-stage univariable-plus-LASSO selection procedure may produce unstable coefficients and optimistic performance estimates. Third, the 70:30 data split left only 33 events for model development and 14 events for internal validation. Although bootstrap resampling was used to estimate selected uncertainty intervals, it did not increase the number of observed events available for fitting or evaluating the model. Fourth, risk thresholds and reclassification measures were exploratory, and no external or prospective validation was performed. Fifth, prophylaxis practices and perioperative care may differ across institutions. The model and web calculator (21) therefore require recalibration, external validation, and clinical-impact evaluation before use in patient care.

## Conclusion

A logistic regression model combining preoperative age-adjusted D-dimer positivity with serum albumin, ADL score, and MMSE score showed preliminary ability to stratify postoperative DVT risk after TURP. Its TURP-specific integration of geriatric domains is clinically relevant, but the model remains exploratory. Prospective multicenter external validation is required before the nomogram, risk strata, or web calculator can inform clinical decisions.

## Data Availability

The raw data supporting the conclusions of this article will be made available by the authors, without undue reservation.
