# Supplementary material for: Geriatric assessment–integrated prediction model for postoperative deep vein thrombosis in older adults undergoing transurethral resection of the prostate: retrospective cohort study
Source: Front Med (Lausanne). 2026 Aug 6;13:1899303. doi: 10.3389/fmed.2026.1899303 (PMC13489928; doi:10.3389/fmed.2026.1899303)
Supplement: Supplementary file 1 [file Supplementary_file_1.docx]

**Supplementary Tables**

**Supplementary Table 1. Performance and quantitative clinical utility of the clinical baseline model**

| Dataset | AUC  (95% CI) | Brier score | Sensitivity | Specificity | AUDC | NB @ 20% |
| --- | --- | --- | --- | --- | --- | --- |
| Development cohort | 0.701  (0.610–0.789) | 0.091 | 0.000 | 1.000 | 0.010 | 0.024 |
| Internal validation cohort | 0.705  (0.561–0.835) | 0.091 | 0.000 | 1.000 | 0.010 | 0.021 |

**Note:** 95% CIs were estimated using 1,000 bootstrap resamples. The clinical baseline model includes preoperative age-adjusted D-dimer positive status only.

**Abbreviations:** AUC, area under the ROC curve; CI, confidence interval; AUDC, area under the decision curve; NB, net benefit.

**Supplementary Table 2. Uniformly calibrated performance of the three integrated models in the internal validation cohort**

| Model | ROC-AUC (95% CI) | PR-AUC | Brier score | Calibration intercept | Calibration slope | NB @ 10% | NB @ 20% |
| --- | --- | --- | --- | --- | --- | --- | --- |
| Integrated LR | 0.751  (0.582–0.914) | 0.467 | 0.082 | 0.382 | 1.294 | 0.035 | 0.046 |
| Integrated RF | 0.774  (0.614–0.910) | 0.472 | 0.085 | 1.357 | 1.870 | 0.037 | 0.046 |
| Integrated SVM | 0.796  (0.653–0.919) | 0.437 | 0.086 | 1.223 | 1.858 | 0.050 | 0.038 |

**Note:** LR, RF, and SVM used the same five-fold development-set cross-validated sigmoid calibration procedure. Balanced class weights were applied only to RF and SVM. The internal validation cohort was used only for final evaluation. Calibration intercept and slope were estimated by regressing the observed outcome on the logit of predicted probability. The paired bootstrap AUC difference between SVM and LR was 0.045 (95% CI: -0.004 to 0.102; P = 0.071).

**Abbreviations:** ROC-AUC, area under the receiver operating characteristic curve; PR-AUC, area under the precision-recall curve; CI, confidence interval; LR, logistic regression; RF, random forest; SVM, support vector machine; NB, net benefit.

**Supplementary Table 3. Diagnostic performance and resource utilization of traditional and age-adjusted postoperative D-dimer strategies**

| Metric | Traditional (>0.50 mg/L) | Age-adjusted | P value / relative change |
| --- | --- | --- | --- |
| Sensitivity | 1.000 | 0.929 | >0.99 |
| Specificity | 0.732 | 0.973 | <0.001 |
| PPV | 0.318 | 0.813 | — |
| NPV | 1.000 | 0.991 | — |
| Ultrasound requirement | 44 (34.9%) | 16 (12.7%) | Relative reduction: 63.6% |

**Note:** The traditional strategy referred patients for lower-limb venous ultrasonography when postoperative D-dimer exceeded 0.50 mg/L. The age-adjusted strategy used Age × 0.01 mg/L for patients older than 50 years. The traditional strategy yielded TP/FP/TN/FN = 14/30/82/0, whereas the age-adjusted strategy yielded 13/3/109/1. This postoperative diagnostic-triage analysis is separate from the preoperative prediction model.

**Supplementary Table 4. Threshold-specific classification performance of the primary integrated logistic regression model in the internal validation cohort**

| Threshold | Cutoff | TP/FP/TN/FN | Sensitivity | Specificity | PPV | NPV | F1-score | Balanced accuracy |
| --- | --- | --- | --- | --- | --- | --- | --- | --- |
| Fixed 0.50 | 0.500 | 1/1/111/13 | 0.071 | 0.991 | 0.500 | 0.895 | 0.125 | 0.531 |
| Development OOF | 0.145 | 9/22/90/5 | 0.643 | 0.804 | 0.290 | 0.947 | 0.400 | 0.723 |
| Risk 0.20 | 0.200 | 8/10/102/6 | 0.571 | 0.911 | 0.444 | 0.944 | 0.500 | 0.741 |
| Risk 0.10 | 0.100 | 9/37/75/5 | 0.643 | 0.670 | 0.196 | 0.938 | 0.300 | 0.656 |

**Note:** The primary integrated LR model used native probabilities from the final logistic regression equation. Its validation ROC-AUC was 0.750, PR-AUC was 0.457, and Brier score was 0.081. The 0.50, 0.20, and 0.10 cutoffs were fixed evaluation thresholds. The 0.145 cutoff was selected by maximizing Youden’s index using out-of-fold predictions confined to the development cohort and is reported as exploratory. TP/FP/TN/FN denotes true positives, false positives, true negatives, and false negatives.

**Abbreviations:** LR, logistic regression; OOF, out-of-fold; PPV, positive predictive value; NPV, negative predictive value; ROC-AUC, area under the receiver operating characteristic curve; PR-AUC, area under the precision-recall curve.

**Supplementary Table 5. Missing-data accounting before construction of the final analytic cohort**

| Item | n/N (%) | Handling |
| --- | --- | --- |
| Incomplete preoperative GA data among screened patients | 21/846 (2.5%) | Excluded before modeling; not imputed |
| Unavailable postoperative ultrasound outcome among screened patients | 163/846 (19.3%) | Excluded before modeling; outcome not imputed |
| Model predictors in final analytic cohort | 0/420 (0.0%) | No imputation required |
| Postoperative ultrasound outcome in final analytic cohort | 0/420 (0.0%) | No imputation required |

**Note:** Percentages before modeling use the 846 screened patients as the denominator. The final analytic cohort was a complete-case cohort; selection bias remains possible.
