# Supplementary material for: Geriatric assessment–integrated prediction model for postoperative deep vein thrombosis in older adults undergoing transurethral resection of the prostate: retrospective cohort study
Source: Front Med (Lausanne). 2026 Aug 6;13:1899303. doi: 10.3389/fmed.2026.1899303 (PMC13489928; doi:10.3389/fmed.2026.1899303)
Supplement: Supplementary file 2 [file Supplementary_file_2.docx]

**Supplementary Figures**

**Supplementary Figure 1**

**Supplementary Figure 1. Corrected nomogram for the primary integrated logistic regression model.** The nomogram is based on the fitted coefficients for preoperative age-adjusted D-dimer positivity (β = 1.641), serum albumin (β = -0.013), ADL score (β = -0.045), and MMSE score (β = -0.106). It is provided as a transparent research-use representation and requires external validation before clinical application.

**Supplementary Figure 2**

**Supplementary Figure 2. Revised research-use interface specification for individualized DVT risk estimation.** Age and preoperative D-dimer are used to derive age-adjusted D-dimer status; serum albumin, ADL score, and MMSE score complete the four-predictor model. Postoperative D-dimer is not a model input. The interface does not provide anticoagulation recommendations and is not intended for treatment decisions.

**Supplementary Figure 3**

**Supplementary Figure 3. Diagnostic performance of traditional and age-adjusted postoperative D-dimer triage strategies in the internal validation cohort (n = 126).** The traditional fixed 0.50 mg/L strategy had sensitivity 1.000, specificity 0.732, PPV 0.318, and NPV 1.000. The age-adjusted strategy had sensitivity 0.929, specificity 0.973, PPV 0.813, and NPV 0.991. This postoperative triage analysis is separate from the preoperative prediction model.

**Supplementary Figure 4**

**Supplementary Figure 4. Precision-recall curves for the uniformly calibrated integrated models in the internal validation cohort.** All three algorithms underwent the same five-fold development-set cross-validated sigmoid calibration. PR-AUC was 0.467 for LR, 0.472 for RF, and 0.437 for SVM. The dashed horizontal line indicates the no-skill baseline corresponding to the observed DVT prevalence (0.111).
